# Seamless Coupling of Chemical Glycan Release and Labeling for an Accelerated Protein *N*-Glycan Sample Preparation Workflow

**DOI:** 10.3390/bioengineering10060651

**Published:** 2023-05-26

**Authors:** Mumtaz Kasim, Anja Griebel, Grit Sandig, Robert Höltzel, Akshay Malhotra, Stephan Hinderlich, Volker Sandig, Barbara Müller, Hans Henning von Horsten

**Affiliations:** 1 Life Science Engineering, HTW Berlin-University of Applied Sciences, Wilhelminenhofstr. 75A, 12459 Berlin, Germany; 2 SERVA Electrophoresis GmbH, Carl-Benz-Str. 7, 69115 Heidelberg, Germany; 3 Laboratory for Biochemistry, Department Life Sciences & Technology, Berlin University of Technology, Seestraße 64, 13347 Berlin, Germany; 4 ProBioGen AG, Herbert-Bayer-Straße 8, 13086 Berlin, Germany

**Keywords:** glycoproteins, glycan analytics, *N*-glycans, Hofmann rearrangement, glycan sample preparation, bioprocessing, FACE fluorescence-assisted carbohydrate electrophoresis

## Abstract

Analytical methods fr direct quantitative *N*-glycan analysis require a sequence of sample preparation and clean-up steps that result in reduced glycan recovery. Therefore, we aimed to combine glycan release and labeling steps. Based on the hypothesis that the reaction mechanism for oxidative chemical glycan release comprises a stable glycan isocyanate intermediate, we investigated whether this could be exploited for the in-situ preparation of fluorescent glycan conjugates. ANTS-labeled *N*-glycans were derived from chicken ovalbumin via an in-situ chemical release/coupling approach and by standard Peptide-*N*-Glycosidase F (PNGase F) digestion/reductive amination. Synoptic fluorescence-assisted carbohydrate electrophoresis with UV detection (FACE-UV) analysis yielded matching patterns of fluorescent *N*-glycan bands in the expected electrophoretic mobility range between hexose units GU-5 and GU-11 of the standard. Anthranilamide (2-AB)-glycan conjugates prepared from a test glycoprotein carrying a predominant Core-F glycan gave single predominant peaks in hydrophilic interaction chromatography with fluorescence detection (HILIC-FLD) and electrospray ionization mass spectrometry (ESI-MS) spectra in agreement with sodiated triply charged Core-F-AB conjugates for both the standard and the in-situ coupling methods. The Core-F-AB conjugate prepared by the in-situ coupling approach had a slightly elevated retention time on HILIC-FLD and an ESI-MS *m*/*z* peak in line with a urea-bonded glycan-AB conjugate, with closed pyran ring structures on the glycan moiety. Glycan isocyanates intermittently formed during chemical glycan release, which could be utilized to prepare labeled glycan samples directly from glycoproteins and fluorescent dyes bearing a primary amine functional group.

## 1. Introduction

*N*-Glycosylation is the most complex post-translational modification of secreted proteins and membrane protein ectodomains. Abnormal variation in glycosylation patterns is linked to human diseases and, therefore, is a potentially valuable biomarker, particularly for early-stage diagnostics. Electrophoretic and HPLC-based analytical techniques have emerged as the methods of choice for quantitative *N*-glycan profiling as they allow for a robust, direct analysis of various oligosaccharide analytes. However, free oligosaccharides cannot easily be detected with sufficient sensitivity by standard analytical detection modes such as FACE-UV, capillary electrophoresis with UV detection (CE-UV), or HPLC-UV or HPLC-RID detection. Instead, oligosaccharide samples must be derivatized with a fluorescent dye to allow for their detection with the required sensitivity. In line with the above, FACE-UV, capillary electrophoresis with laser-induced fluorescence (CE-LIF), and HILIC-FLD are well-established methods for the quantitative analysis of labeled glycans. Fluorescently labeled glycan samples prepared by current labeling strategies are detectable at the femtomolar range [1] and very well suited for quantitative glycan analysis since there is a direct correlation between peak fluorescence intensity and the relative abundance of the resolved labeled glycan species [2]. The capability of these methods to robustly and reliably quantify incurred glycan samples has been attributed to the fact that only one fluorophore molecule is stoichiometrically added per each individual glycan reducing end [2,3].

Unfortunately, the need for the fluorescence labeling of glycan samples comes at the expense of added sample preparation steps that contribute to a lowered overall sample recovery and increased sample processing timeframes. Originally, enzymatically re-leased *N*-glycans were derivatized with a fluorescent label by reductive amination, with compounds comprising a primary amine group capable of forming a Schiff-base, with the aldehyde functional group at the reducing end of the glycan [4]. More recent approaches to minimize the required time needed for the preparation of labeled glycan samples are based on fluorescent dyes carrying an *N*-hydroxysuccinimide carbamate, which rapidly modifies glycosylamine-bearing *N*-glycans. The latter occurs as an intermediate after their enzymatic release by Peptide-*N*-Glycosidase F (PNGase F), a broad specificity amidase [5,6]. The coupling of *N*-glycan glycosylamines to *N*-hydroxysuccinimide carbamate fluorescent dyes results in stable fluorescent glycan conjugates carrying a stable urea linkage [5,6]. The preparation of fluorescent glycan samples by carbamate-glycosylamine coupling directly proceeds in an aqueous solution and thereby obviates lengthy drying steps and acidic reaction conditions that may potentially cause the degradation of precious glycan samples. Importantly, it must be noted that glycosylamine intermediates are unstable in aqueous solutions and must thus be reacted with the carbamate dye within a timeframe of 5 to 10 min [6]. Therefore, this labeling approach requires the conjoined use of a fast-acting PNGase F.

Although the current glycan sample preparation workflow based on carbamate/glycosylamine coupling enables the omission of a first glycan clean-up step, it is still dependent on expensive reagents and proprietary enzymes that drive up the cost per measure point in routine analysis. Therefore, we addressed the possibilities to employ chemical methods as a means of combining glycan release and fluorescent labeling. The authors of the most recently published method for chemical glycan release by hypochlorite proposed a mechanism reminiscent of the classical Hoffmann rearrangement of carboxamides [7]. This well-known reaction results in the intermittent formation of a highly reactive isocyanate that could either be subject to hydrolysis or to stable carbamate- and urea-bonding to different nucleophilic reagents bearing hydroxy-groups or aliphatic and aromatic primary amines [4]. Therefore, we hypothesized that this intermittently occurring isocyanate could be exploited to accomplish the simultaneous release of glycans and the coupling of the nascent glycan isocyanate to a nucleophilic fluorescent amine moiety via a stable urea bond.

Here, we report an optimized method for simultaneous one-pot chemical *N*-glycan release and fluorescent labeling of the *N*-glycan species for seamless subsequent sensitive detection. In addition, we also report that subsequent FACE analysis in ultrathin polyacrylamide gels has the capacity to resolve labeled glycans in the presence of large amounts of excess label, thereby obviating the need for additional clean-up steps.

## 2. Materials and Methods

### 2.1. Materials

Chemicals and solvents were obtained from Serva Electrophoresis GmbH (Heidelberg, Germany), Merck KGaA (Darmstadt, Germany), Sigma Aldrich (St. Louis, MO, USA), Acros Organics (Geel, Belgium), Carl Roth (Karlsruhe, Germany), or Thermo Fisher Scientific Waltham, MA, USA). All electrophoresis reagents and supplies were purchased from SERVA Electrophoresis GmbH (Heidelberg, Germany). Hypochlorite solution was purchased from CP GABA GmbH (Hamburg, Germany; 2.8% NaClO) and diluted before use. Solid phase extraction cartography columns were purchased from Sigma Aldrich (St. Louis, MO, USA). C18-based SPE mini-columns (SPE-column, C18 end-capped, 750 mg/6 mL) were ordered from Applichrom GmbH, (Oranienburg, Germany).

### 2.2. Enzymatic Release of N-Glycans

*N*-glycans were released by PNGase F according to the vendor’s instruction manual.

### 2.3. Fluorescent Labeling of N-Glycans and Dextran Hydrolysate Standards

*N*-glycans were labeled with 2-aminobenzamide (2-AB) according to a protocol modified from that described by Ruhaak et al. [2]. A total of 100 µL of an aqueous solution of glycans was incubated at 65 °C for 1 h with 50 µL 2-AB (48 mg/mL stock in DMSO), 50 µL freshly prepared 1 M sodium cyanoborohydride (DMSO), and acetic acid at a final concentration of 15% (*v*/*v*). After completion of the incubation time, the reaction was stopped by adding 50 µL 100% acetonitrile. The excess label was removed by passing through a C18-based SPE mini-column (Applichrom GmbH). A 20% acetonitrile/0.1% formic acid mobile phase was applied at a flow rate of 0.5 mL/min. The eluted glycans were 85–95% pure from excess label.

In order to label glycans with 8-aminonaphthalene-1,3,6-trisulfonic acid (ANTS), all glycan standards and samples needed to be dried completely. The dried glycan samples were then redissolved in 5 µL of 0.1 M ANTS (in 15% acetic acid in water), and 5 µL 1 M sodium cyanoborohydride (DMSO) was added. The reaction mix was then incubated for 16 h at 37 °C. After completion of the ANTS labeling reaction, samples were ready to be analyzed by fluorescence-assisted carbohydrate electrophoresis on an HPE Blue Horizon flatbed electrophoresis system (BHZ-FACE).

### 2.4. In Situ Coupled Chemical N-Glycan Release and Labeling

A total of 100 µL of glycoprotein (20 mg/mL) was placed on ice and mixed with 100 µL of a borax solution (saturated at RT); 200 ul of an ice-cold hypochlorite solution (% *w/v* as indicated in the text) was then added and the mix was incubated for 2 min with shaking at RT. A fluorescent label bearing a primary amine (i.e., 50 µL 0.1 M ANTS in water or 50 µL 2-AB (50 mg/mL in DMSO)) was then added and the mix was incubated for 10 min at 37 °C with shaking. A total of 20 µL formic acid was then added to stop the reaction and the mix was placed on ice for 2 min. Finally, any residual particulate matter was removed from the mix by spinning the reaction vial for 2 min at 10,000 g at RT. The clarified supernatant was diluted 10-fold with distilled water prior to purification on a cartography SPE column. Prior to use, the column was activated with 3 mL of 100% acetonitrile, followed by a wash with 4 mL of distilled water. The diluted sample was loaded, washed briefly with 2 mL water, and eluted in 0.5–1 mL 50% acetonitrile. The glycan sample was dried and resuspended in 20 µL water for ANTS-labeled samples or 20 µL HILIC solvent (60% acetonitrile (*v*/*v*) in 10 mM ammonium formate buffer pH 4.4).

### 2.5. Preparation of BHZ-FACE Glycan Electrophoretic Mobility Standard

ANTS-labeled dextran partial hydrolysate was used as the standard for assessing the electrophoretic mobility of different ANTS-labeled hexose units. This standard was prepared as described previously [8]. Briefly, 100 mg of dextran (Cat.-No. 31398; molecular weight ~200,000, (Sigma Aldrich (St. Louis, MO, USA) was suspended in 10 mL of 0.1 N HCl and incubated at 100 °C for 4 h. Then, the solution was blow-dried in a nitrogen evaporator. The dried carbohydrate sample was suspended in 500 µL each of 0.2 M ANTS in acetic acid–water (3:17, vol/vol) and freshly made 1.0 M sodium cyanoborohydride in dimethyl sulfoxide and incubated at 37 °C for 16 h. The samples were then dried in a fume hood under nitrogen at 45 °C and stored at −20 °C. The standard was dissolved in a GlycoGel loading buffer (Serva Electrophoresis GmbH, Heidelberg, Germany) prior to use.

### 2.6. Blue Horizon Horizontal FACE Gel Electrophoresis (BHZ-FACE)

ANTS-labeled glycan samples were separated by fluorescence-assisted carbohydrate electrophoresis on ultrathin, foil-supported GlycoGels that were run in an HPE™ BlueHorizon™ flatbed chamber (Serva Electrophoresis GmbH, Heidelberg, Germany) (BHZ-FACE), according to a published protocol [9].

### 2.7. Preparation of Recombinant RSVF Protein from SF-9 Insect Cells

The cDNA encoding the His-tagged RSVF-ectodomain glycoprotein was constructed as described earlier [10]. Briefly, an RSVF cDNA construct consisted of the coding sequence for a melittin signal sequence (Acc. No. P01501; MKFLVNVALVFMVVYISYIY) followed by the ectodomain of the RSVF protein (amino acids 26 to 530; Acc. No. EF566942), a Factor Xa cleavage site (IEGR), and a GSGS linker fused to a 6×His-tag (HHHHHH). This recombinant transgene was synthesized with flanking EcoRI and BamHI endonuclease cleavage sites and cloned into the pFASTBAC vector, as described earlier [11]. The assembled vector construct was transformed into DH10Bac *E. coli*-competent cells in order to form the infectious recombinant baculovirus expression bacmid via recombination of the RSVF encoding transgene cassette from the pFASTBAC vector with the parent bacmid from the DH10Bac host cells. The recombined bacmid was used to generate initial virus titer by infecting Sf-9 insect cells. The recombinant baculovirus was plaque purified, expanded, titrated by TCID_50_ endpoint dilution assay, and used to infect Sf-9 lepidopteran insect cells at an MOI of 5. A total of 72 h post-infection, the supernatant was clarified and the buffer was exchanged with a bind/wash buffer (20 mM sodium phosphate, 0.5 M NaCl, 20 mM imidazole, pH 7) via tangential flow filtration using a Minimate TFF Capsule w/10 Kd Omega (Pall Corporation, Port Washington, NY, USA).The recombinant RSVF protein was then purified from the buffer-echanged supernatant by Nickel-NTA-IMAC on a 5 mL NTA cartridge (Machery & Nagel, Düren, Germany). The column was pre-equilibrated with 5 volumes of bind/wash buffer and then the rebuffered cell culture supernatant was loaded onto the column. After washing the column with 10 volumes of bind/wash buffer, the protein was eluted by step elution with a 5 mL elution buffer (20 mM sodium phosphate, 0.5 M NaCl, 500 mM imidazole, pH 7). The eluate was then rebuffered against PBS by dialysis, filtered through a 0.45 µm sterile filter, and stored at 4 °C. Protein concentration in the rebuffered eluate was then quantified by BCA assay for the determination of the total protein concentration. Finally, the eluted protein fraction was resolved on 12% SDS-PAGE with silver staining to confirm the identity of the expressed RSVF glycoprotein.

### 2.8. High-Performance Liquid Chromatography (HPLC)

2-anthranilamide (2-aminobenzamide, 2-AB)-labeled glycan samples were analyzed by hydrophilic interaction liquid chromatography with fluorescence detection (HILIC-FLD) on an OTU-amino column (105 Å, 5 µm, 250 mm × 4.6 mm; Applichrom GmbH, Oranienburg, Germany), as previously described [8]. A fluorescence excitation at 360 nm and emission at 428 nm was used to detect 2-AB glycans and sugar standards. The HILIC column temperature was set at 40 °C and the column was operated at a flow rate of 1.0 mL/min in isocratic elution mode with 60% acetonitrile in a 10 mM ammonium formate buffer (pH 4.4).

### 2.9. Electrospray Ionization Mass Spectrometry (ESI-MS)

A Varian-500 MS system was used to record mass spectra for 2-AB-labeled glycan samples. A total of 5.0 kV was applied to the MS inlet in positive ion mode. The sample peak was collected from HILIC runs conducted with a mobile phase containing LC/MS-grade acetonitrile. A flow rate setting of 8 µL/min was used to infuse the glycan samples into the ESI source. Settings for nitrogen drying gas temperature and pressure were 250 °C and 10 psi. The nitrogen-nebulizing gas pressure was set to 35 psi. The helium damper gas (helium) was set at a flow rate of 0.8 mL/min. Spectra were acquired within a mass range of *m*/*z* 400–1000. The tuning mix solution Varian/Agilent was infused at a flow rate of 10 μL/min for the external calibration of the ESI-MS unit. For sample analysis in standard MS/MS mode, glycan samples were infused at a flow rate setting of 4 μL/min. Glycans were identified based on the accurate mass of doubly and triply charged glycans (mass deviation <2 ppm). Data analysis was performed using the Varian Workstation (now Agilent).

## 3. Results

### 3.1. Parallel Analysis of Multiple Glycan Samples on Blue Horizon Foil-Supported Ultrathin FACE Gels (BHZ-FACE)

**Figure 1:**
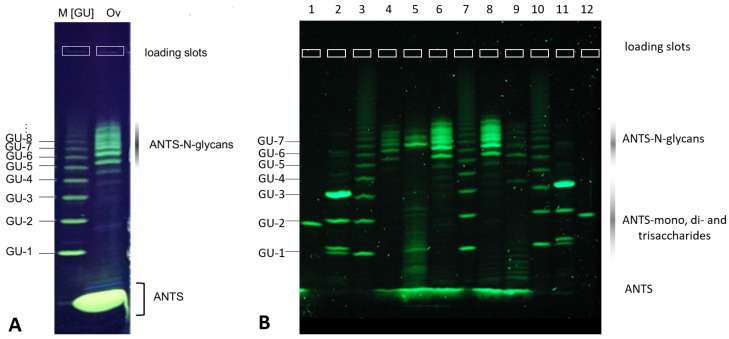
Parallel synoptic analysis of multiple ANTS-labeled glycan samples by BHZ-FACE. (**A**) Concomitant BHZ-FACE separation and UV detection of resolved ANTS-glycan bands for the dextran standard and *N*-glycans derived from chicken ovalbumin. Note the excellent ability of the method to resolve samples in the presence of a major amount of excess label. (**B**) Simultaneous and parallel separation and detection of multiple ANTS-glycan samples. Lanes 1 and 12: ANTS-lactose; lanes 2 and 11: mix of ANTS-mannose, -maltose, and -maltotriose; lanes 3, 7, and 10: ANTS-labeled dextran standard, lanes 4 (1 µL), 6 and 8 (2 µL): ovalbumin *N*-glycans (different concentrations), lane 9: alpha-1-antitrypsin *N*-glycans; lane 5: IgG1-Fc glycans. FACE-system: HPE™ BlueHorizon™, SERVA HPE™ GlycoGel (125 × 250 mm × 0.43 mm).

Method development for glycan sample preparation workflows depends on the ability to comparatively analyze multiple samples from many different scouting runs. Analytical methods based on HPLC and capillary electrophoresis (CE) inherently need to run consecutively, which causes extended run times for multiple samples as well as comparatively lower within-run precision. In order to obtain a synoptic read-out from scouting experiments in method development for the concurrent glycan release and labeling technique, we first developed a robust analytical electrophoretic separation method for ANTS-labeled glycans in ultrathin, foil-supported, horizontal polyacrylamide gels. An ANTS-labeled dextran standard resolved different ANTS-labeled glycan bands in a range between 1 and 13 glucose units ([GU]) (Figure 1A, lane M[GU], Figure 1B lanes 3, 7, and 10). BHZ-FACE resolved ANTS-labeled *N*-glycans from chicken ovalbumin, even in the presence of a major excess of unconjugated ANTS label (Figure 1A, lane Ov). Figure 1B shows the parallel electrophoretic analysis of 12 ANTS-labeled glycan samples on a BHZ-FACE GlycoGel (Serva Electrophoresis GmbH). ANTS- mono-, di- and trisaccharides were well separated from excess label and resolved in agreement with the respective dextran standard GU units (Figure 1B, lanes 1, 2, 11, 12). ANTS-labeled *N*-glycans derived from chicken ovalbumin and IgG1-Fc yielded fluorescent band patterns in the expected range between GU-5 and GU-11 of the dextran standard (Figure 1B, lanes 4, 5, 6, 8, 9). As expected, chicken ovalbumin and IgG1-Fc yielded different *N*-glycan profiles on BHZ-FACE. The three observed predominant glycan bands from the IgG1-Fc sample represent the expected G0F, G1F, and G2F glycan structures, in agreement with their expected relative abundance (Figure 1B, lane 5) (Core-F = basic *N*-glycan structure consisting of two glycosidically linked *N*-acetylglucosamine moieties at the reducing end, a fucose monosaccharide linked via an alpha-1,6-glycosidic bond to the proximal *N*-acetylglucosamine, and a branching trimannose structure at the nonreducing end; G0F, G1F, G2F = biantennary Core-F type glycan structures carrying *N*-Acetylglucosamine on each terminating mannose of the trimannosyl core, followed by either 0, 1, or 2 galactose moieties).

The developed BHZ-FACE technology was subsequently applied as a tool for screening and method development in the discovery of optimized conditions for the one-pot chemical glycan release and fluorescent labeling strategy.

### 3.2. Development and Optimization of a Method for the Seamless Coupling of Chemical Glycan Release and Fluorescent Labeling

**Figure 2:**
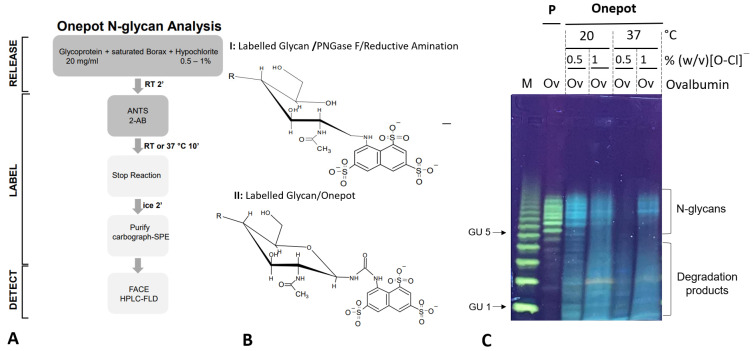
Seamless coupling of chemical glycan release and fluorescent labeling. (**A**): Schematic representation of the developed optimized workflow for seamless chemical glycan release and labeling with fluorescent compounds bearing a primary amine (one-pot *N*-glycan analysis). (**B**): Chemical structures of the ANTS-conjugated *N*-glycans prepared by the benchmark PNGaseF/reductive amination labeling approach (I) and the one-pot *N*-glycan coupling described here (II). The benchmark method produced fluorescent glycan conjugates with an open pyran ring structure at the proximal glycan end linked to ANTS via a reduced Schiff base linkage. The one-pot method produced stable urea-bonded ANTS-glycan conjugates with a closed pyran ring structure at the proximal end. (**C**): Synoptic BHZ-FACE of chicken ovalbumin glycan samples prepared by the benchmark method and different one-pot reaction conditions. M: ANTS-labeled dextran standard; P: Chicken ovalbumin ANTS-*N*-glycans prepared by PNGaseF/reductive amination (benchmark sample); one-pot lanes: chicken ovalbumin ANTS-*N*-glycan samples prepared by the one-pot approach under the conditions indicated; GU: glucose units of the dextran standard; [O-Cl^−^]: hypochlorite concentration; ANTS: 8-amino-naphthalene-1,3,6-trisulfonate.

The BHZ-FACE methodology described in 3.1 enabled us to directly analyze and compare fluorescent glycan samples from multiple preparation runs and conditions. Using the *N*-glycosylated glycoprotein chicken ovalbumin as a test item and BHZ-FACE-aided glycan profiling as a read-out for method development, we identified the optimum conditions for a seamlessly coupled sample preparation workflow that yields fluorescently labeled *N*-glycan samples (Figure 2A). For the initial method development, reaction conditions of 20 °C and 37 °C and 0.5% and 1% (*w/v*) hypochlorite ([OCl^−^]) were screened (Figure 2C). Except for the 37 °C, 0.5% [OCl^−^] setting, fluorescent bands were observed in the relevant range for ANTS-labeled *N*-glycans between GU-5 and GU-15 of the dextran standard for electrophoretic mobility (Figure 2C). The optimum conditions reflected by the strongest relative fluorescence intensity of the identified band pattern within the *N*-glycan range were observed for the 20 °C, 0.5% [OCl^−^] setting (Figure 2C). A synoptic comparison of the ANTS-labeled ovalbumin *N*-glycans from the coupled glycan sample preparation (Onepot) and the ANTS-labeled ovalbumin *N*-glycans prepared by the benchmark method (P, PNGaseF release/reductive amination coupling) revealed distinct fluorescent bands with a slightly shifted electrophoretic mobility for these two samples (Figure 2C, lanes P and lane Onepot 20 °C/0.5% [OCl^−^]).

### 3.3. Preparation of a Homogeneously Glycosylated Test Glycoprotein

**Figure 3:**
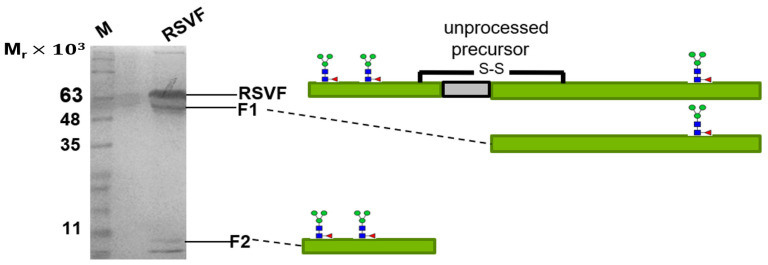
SDS-PAGE separation of purified recombinant RSVF ectodomain (reducing conditions). Recombinant RSVF glycoprotein derived from baculovirus-infected Sf-9 insect cells was separated on 12% SDS-PAGE under reducing conditions and silver-stained. The observed bands reflected the expected polypeptides resulting from incomplete differential proteolytic processing of the precursor at two furin cleavage consensus sites.

In order to confirm the identity of the chemical structure of the conjugated glycans resulting from the one-pot approach, we needed a test glycoprotein carrying a homogeneous *N*-glycan structure. To this end, we obtained a recombinant baculovirus, encoding a HIS-tagged version of the respiratory syncytial virus F-protein (RSVF) [11]. Previous data had shown that this SF—derived RSVF protein contained three occupied *N*-glycosylation sequons, each bearing a homogeneous paucimannosidic *N*-glycan structure of the Core-F type [11]. The Sf-9-derived recombinant RSVF ectodomain resolved on SDS-PAGE into three distinct bands resulting from differential proteolytic processing (Figure 3).

### 3.4. Comparative Analysis of 2-AB-Labeled Glycans Derived from PNGaseF/Reductive Amination Labeling and Chemical One-Pot Release/Coupling

**Figure 4:**
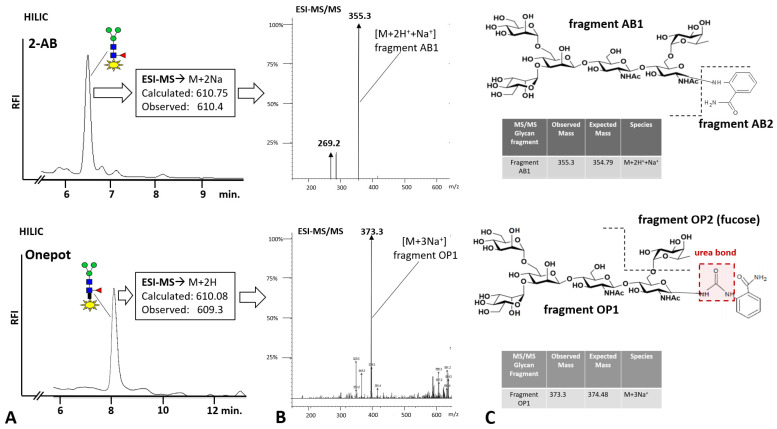
Comparative analysis of 2-AB RSVF Core-F glycans from the one-pot and PNGaseF/reductive amination approaches. (**A**) HILIC analysis of 2-AB-labeled RSVF glycans prepared by PNGaseF release and reductive amination (2-AB, chromatogram top left) and one-pot-derived RSVF glycans labeled with 2-AB (Onepot, chromatogram, bottom left). HILIC conditions: column: OTU amino (105 Å, 5 µm, 250 mm × 4.6 mm), column temperature: 40 °C, eluent: isocratic 60%ACN, 10 mM ammonium formate, pH 4.4, flow rate 1 mL/min.; detection via Jasco FP 1520 with fluorescence excitation of 360 nm and emission of 428 nm. A single predominant peak was observed in both comparative runs. Peak retention volume was shifted to an extended range for the one-pot-derived sample. (**B**) ESI-MS/MS data for the two sample peaks. The benchmark sample yielded an MS/MS spectrum with a predominant peak at *m*/*z* 355.5. The one-pot sample produced a prominent peak at *m*/*z* 373.3 in the MS/MS spectrum. (**C**) Annotation of MS/MS peaks: The peak at *m*/*z* 355.5 from the MS/MS spectrum of the benchmark sample was consistent with a protonated and sodiated triply charged fragment AB1 resulting from cleavage of the anthranilamide moiety. The peak at *m*/*z* 373.3 from the MS/MS spectrum of the one-pot sample was consistent with a sodiated triply charged fragment OP1 resulting from cleavage of the fucose moiety.

Sf-9-derived RSVF glycoprotein from Section 3.3 was used as a test item to prepare 2-AB-labeled *N*-glycan samples by the benchmark and one-pot methods. The traditional benchmark method consists of PNGase F-mediated glycan release and subsequent labeling via reduced Schiff-base formation, resulting in a single prominent peak detected by HILIC-FLD (Figure 4A, top left). This peak gave a clean ESI-MS peak at *m*/*z* 610.4, which is consistent with a doubly charged sodiated species. Subsequent MS/MS yielded a prominent peak at *m*/*z* 355.5 (Figure 4B, top), which is consistent with a sodiated triply charged fragment AB1 (Figure 4C, top right) derived from the doubly charged precursor detected in ESI-MS. The one-pot chemical glycan release and coupling method resulted in a single prominent peak detected by HILIC-FLD (Figure 4A, bottom left). Compared to the 2-AB glycan peak obtained by the benchmark method, this peak had a slightly extended elution volume on HILIC. ESI-MS yielded a peak at *m*/*z* 609.3, which is consistent with a doubly charged protonated species. Subsequent MS/MS of this precursor ion yielded a prominent peak at *m*/*z* 373.3 (Figure 4B, bottom), which is consistent with a sodiated triply charged fragment OP1 (Figure 4C, bottom right). HILIC-FLD and ESI-MS data for the glycan AB conjugate obtained by one-pot coupling identified this molecule as an AB-labeled glycan urea bonded to the proximal glycan moiety via the aromatic primary amine of the anthranilamide (2-AB). Interestingly, the two different glycan conjugates appeared to fragment differentially in ESI-MS/MS. While the traditional 2-AB glycan conjugate was cleaved at the bond linking the glycan moiety to the 2-AB molecule, the urea-bonded construct was fragmented at the bond linking core fucose to the proximal glycan *N*-acetylglucosamine.

## 4. Discussion

The quantitative analysis of glycans requires the direct detection of glycan analytes from incurred samples. Typically, glycoprotein samples are available in small amounts, which, in turn, results in very small concentrations of prepared glycan analytes that remain below the limit of detection of common HPLC-, CE, and FACE detectors. Therefore, such glycan samples must be derivatized with a fluorescent label to enable their robust detection and raise method sensitivity. Current advanced glycan sample preparation methods offer the possibility to accelerate the sample preparation workflow and reduce hands-on labor. Unfortunately, these glycan sample preparation methods rely on the use of proprietary enzymes and specifically synthesized reactive fluorescent labels, which render these technologies inflexible and ramp up the cost per measure point.

**Figure 5:**
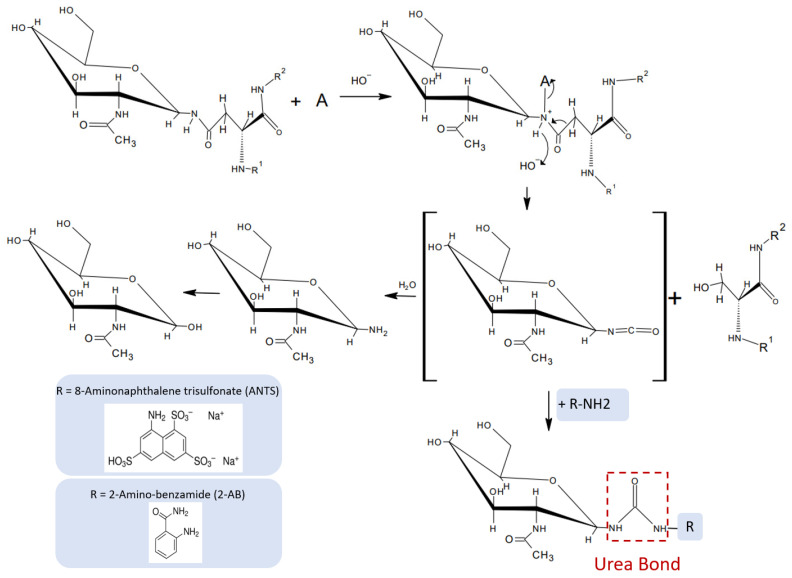
Proposed mechanism for seamless coupling of chemical glycan release and fluorescent labeling (one-pot mechanism).

Based on the mechanism proposed by Song et al. [7] for oxidative chemical glycan release, we hypothesized that hypochlorite-mediated glycan release from an *N*-glycoprotein intermittently produces *N*-glycan-derived glycan-isocyanates that could form urea-bonded conjugates with a fluorescent primary amine (Figure 5). Based on the notion that the reactivity of an isocyanate depends on the type of nucleophile employed in the reaction, we hypothesized that primary amine species would be more reactive towards isocyanate and compete for water in an electrophilic addition reaction with isocyanates. The uncatalyzed rate of the electrophilic addition of isocyanates decreases in the following order: primary aliphatic amines > secondary aliphatic amines > aromatic amines > primary hydroxyls > water. Reactions of isocyanates with amines are extremely fast, with a relative reaction rate 1000 times higher than the reaction rate of isocyanate and water [12], and completely independent of the presence of a catalyst. This important information led us to investigate whether fluorescent detection reagents carrying a primary amine could be utilized to accomplish a one-pot glycan release and coupling of the nascent isocyanate glycan-oligosaccharide to an amine-nucleophilic fluorescent detection label.

To test the hypothesis, we first developed an optimized sample preparation workflow for the one-pot synthesis of ANTS-labeled *N*-glycan conjugates directly from chicken ovalbumin test glycoprotein. Samples were then analyzed in parallel by synoptic BHZ-FACE on foil-supported, ultrathin polyacrylamide gels. Optimized one-pot reaction conditions (Figure 2A) resulted in a pattern of ANTS-*N*-glycans that matched the pattern obtained for ovalbumin *N*-glycans obtained by the traditional workflow (Figure 2C). Interestingly, we observed a slightly shifted electrophoretic mobility for the ANTS-labeled *N*-glycans derived from the one-pot approach (Figure 2C). This may be explained by the different chemical structures of the glycan conjugates (Figure 2B). It has been observed earlier that the HILIC-FLD retention time of labeled *N*-glycans is impacted by the chemical structure of the attached fluorophore [13]. Specifically, urea-bonded *N*-glycan conjugates with closed pyran-ring structures were retained longer on HILIC compared to the respective *N*-glycan conjugates linked by a reduced Schiff base with an open pyran ring [13]. Given the orthogonal comparability between HILIC separation and gel electrophoresis and the fact that carbamate-glycosylamine coupling and the one-pot approach both yielded similar urea-bonded conjugates, these results from method prospecting were very promising. Therefore, we decided to apply the optimized one-pot approach to a test glycoprotein bearing a prominent single homogeneous *N*-glycan. Previously reported data for the Sf-9 insect cell-derived RSVF protein have shown that this protein has three occupied *N*-glycan sequons, each carrying a prominent single homogeneous Core-F type glycan structure, with only minor alternative glycan impurities [11].

**Figure 6:**
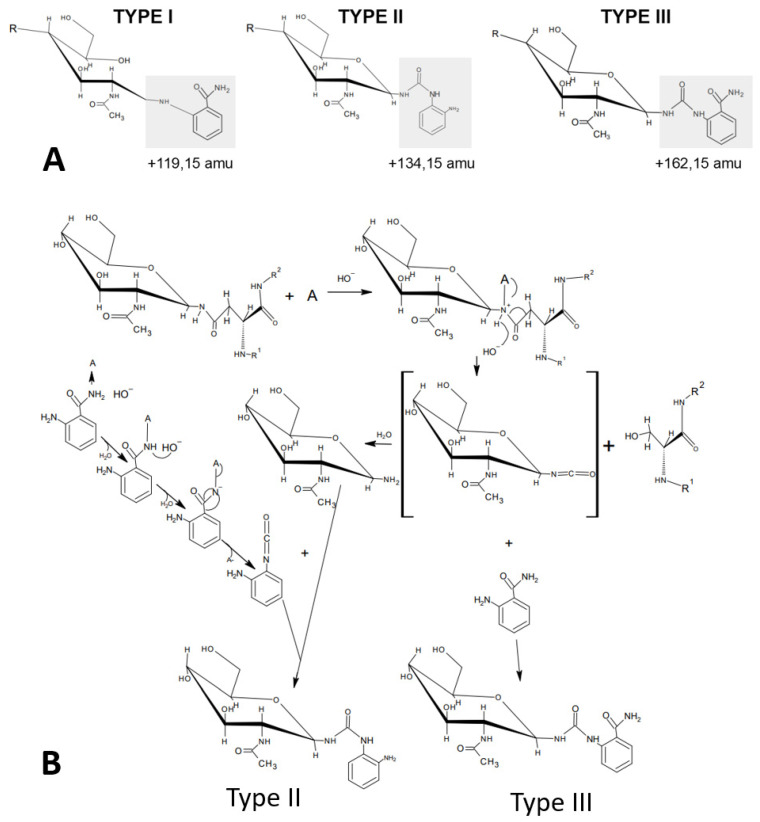
Different possible types of 2AB-glycan conjugates. (**A**) Types of possible 2-AB *N*-glycan conjugates. Type I: reduced Schiff base/open ring (benchmark method); Type II: urea-bonded via 2-AB-derived isocyanate; Type III: urea-bonded via *N*-glycan isocyanate (**B**): Proposed mechanism for the generation of type II and III glycan conjugates. amu: atomic mass units.

To corroborate the findings for chicken ovalbumin on BHZ-FACE with orthogonal methodologies, we then used the RSVF test glycoprotein and anthanilamide (2-AB, aminobenzamide, 2-AB) in a one-pot coupled glycan release reaction (Figure 4). We observed an extended retention time for the one-pot-derived glycan conjugate peak on HILIC-FLD (Figure 4B) compared to the traditional Schiff-base conjugate (Figure 4A). This was in good agreement with published records for the HILIC separation of urea-bonded glycans [13] and our findings for the chicken ovalbumin glycan conjugates resolved on BHZ-FACE (Figure 2C). 8-Amino-napthaline-1,3,6-trisulfonate (ANTS), the fluorescent label used in the BHZ-FACE method scouting, was only able to couple via its aromatic primary amine (Figure 2). Since we had discovered that 2-AB was performing much better in one-pot reactions compared to anthranilic acid (2-AA), we realized that the carboxamide group of anthranilamide (2-AB) could itself be converted into a reactive isocyanate and, in turn, couple to a glycosylamine to form type II urea-bonded glycan conjugates (Figure 6).

Therefore, in order to confirm that 2-AB was indeed coupled by the same mechanism, we analyzed the eluted peaks from HILIC by ESI-MS/MS and confirmed the linkage of the respective glycan conjugate as a type III urea-bonded species (Figure 4B,C) that had resulted from the coupling of the intermittently occurring glycan isocyanate to the aromatic primary amine of 2-AB. The prominent ESI-MS/MS fragment resulting from the Schiff base-linked glycan-AB conjugate at *m*/*z* 355.3 (Figure 4B, top) represented a triply charged sodiated species of fragment AB-1 (Figure 4C, top) that had resulted from the fragmentation of the precursor at the reduced Schiff base linkage. For the glycan-AB conjugate that had been generated in the one-pot reaction, a prominent fragment peak at 373.3 *m*/*z* was observed, which indicated the fragmentation of the urea-bonded conjugate precursor at the linkage between fucose and the proximal *N*-actetylglucosamine moiety. These findings show that one-pot conjugation of protein-bound glycans is mediated by a type III urea bond and may also indicate a higher stability of the urea bond compared to the reduced Schiff base linkage.

The predominant formation of a type III conjugate by 2-AB one-pot glycan coupling is also supported by the literature on isocyanate chemistry. Theoretically, an aromatic isocyanate such as the product resulting from the Hofmann carboxamide rearrangement of anthranilamide (2-AB) should have a stronger reactivity towards nucleophilic reagents since an increase in the positive charge on the carbon of the isocyanate group facilitates the nucleophilic addition of amines and hydroxylic compounds [14]. Aromatic isocyanate compounds have a stronger positive charge on the isocyanate carbon atom because the negative charge gets delocalized into the aromatic π electron ring system [14]. In turn, this means that aromatic isocyanates are more reactive than aliphatic or cycloaliphatic isocyanates [14]. On the other hand, isocyanate reacts with water to form an unstable carbamic acid intermediate that immediately decomposes to amine and CO_2_ [14]. This reaction is typically slow in the absence of a catalyst [14]. In contrast to this, urea bond formation between isocyanate and a primary amine is diffusion-controlled with a significant reaction enthalpy of approximately 40 kcal/mol [15]. In an aqueous solution, the kinetics of urea bond formation are dominated by the reaction of isocyanate and amine when the reaction of isocyanate and water remains uncatalyzed [16]. For the reaction between isocyanate and amine, an apparent first-order kinetic dependence on isocyanate concentration and a second-order dependence on the amine concentration and a relatively low activation energy (~17.5 kJ/mol) have been reported [16]. This accounts for the very high initial reactivity of isocyanate–amine systems [16]. In the presence of an excess of a primary amine-bearing fluorescent label, nascent glycan isocyanates could be expected to react much more quickly with an aromatic primary amine than with water.

## 5. Conclusions

We report a very convenient, flexible, and affordable route towards preparing fluorescently labeled *N*-glycan conjugates amenable to subsequent analysis by suitable analytical separation and detection technologies.

## 6. Patents

von Horsten, H.H., Kasim M., Controlled Release of Glycans from Glycoproteins and Enveloped Viruses, Application Number: PCT/EP2020/079238.

## Data Availability

Data are contained within the article.
